# FIMEC Test to Evaluate the Water Uptake of Coated and Uncoated CFRP Composites

**DOI:** 10.3390/ma13051154

**Published:** 2020-03-05

**Authors:** Francesco David, Patrizia Moretti, Vincenzo Tagliaferri, Federica Trovalusci

**Affiliations:** Dipartimento di Ingegneria dell’Impresa, Università degli Studi di Roma “Tor Vergata”, via del Politecnico 1, 00133 Roma, Italy; francesco.david@uniroma2.it (F.D.); patrizia.moretti@uniroma2.it (P.M.); federica.trovalusci@uniroma2.it (F.T.)

**Keywords:** FIMEC test, water uptake, carbon fiber, thermosetting resin

## Abstract

This study focuses on the application of the FIMEC (flat-top cylinder indenter for mechanical characterization) indentation test to evaluate the effect of water uptake on the mechanical properties of high-performance materials, in particular CFRP (carbon fibre reinforced polymer) composites. Coated and uncoated samples were analyzed. Silicon-based and siloxane coatings were formulated and applied to CFRP to reduce the moisture absorption of the material. The FIMEC test was adopted to study the reduction of the stiffness of CFRP plates for different ageing in water. The evolution of mechanical properties is reported as a function of the water uptake. IR analyses and weight variation measures were used as supporting data. Experimental results show that the FIMEC test is suitable to assess the stiffness reduction due to the aging in water and to identify coatings able to minimize the water uptake.

## 1. Introduction

A non-destructive indentation test, the FIMEC (flat-top cylindrical indenter for mechanical characterization) test, suitable to evaluate the local mechanical properties of CFRP (Carbon Fibre Reinforced Polymer) plates [1], was used in this work to characterize sample and to study the effect of moisture absorption on the stiffness, according to a preliminary study by the authors [2]. The evolution of mechanical properties evaluated by FIMEC is reported as a function of the water uptake.

The water uptake for composite materials is generating more and more interest, in particular for high-performance applications, and many studies have focused on the mechanism and the moisture effect [3,4]. Water uptake may lead to the deterioration of mechanical properties of high strength composites [5,6]. In [7] the authors studied the durability of pultruded CFRP plates under sustained bending strain immersed in seawater and water at room temperature. They concluded that the immersion in both the medium produced the degradation in the resin controlled property (i.e., short beam shear strength) of CFRP. The conclusion showed much less or negligible effects on the fiber controlled properties (i.e., tensile strength and modulus). The same considerations were obtained in [8] from acoustic emission analysis. The authors suggested that matrix interface weakening is the main damage mechanism induced by water ageing for composites.

The water absorption behavior of the matrix itself plays an important role in the water absorption of composites. The chemical composition of matrices (crystallinity and the orientation of polymer molecules) influences the diffusion properties of molecules. The maximum moisture content, M%_max_, is dependent on the polymer chemistry, as reported in [9], with typical values being: M%_max_ < 10% (strongly hydrophilic) for polyvinylalcohol, polyacrylic acid, polyacrylamide; M%_max_ < 7% for cured epoxy; M% _max_ < 0,1% (moderately hydrophilic) for polyethers, polycarbonate, polyesters; and M%_max_ < 0,1% (non-hydrophilic groups) for polyolefins, PTFE, and polydimethylsiloxane. 

To increase the water uptake effect on the composite material and mark the differences between coating conditions, the specimens considered in the present study were made in an epoxy resin matrix. This choice was driven by the resulting high mechanical properties, good chemical resistance, low residual stress, remarkable thermal stability, and excellent ability to adhere to the fibers conferred by the epoxy resin. 

The FIMEC test is already applied in the scientific literature to investigate aluminum alloys [10], steel [11], polymers [12], and composites [13]. As regards the main studies involving indentation tests on polymer matrix composites, local concentration and dispersion of powder fillers can be studied by FIMEC, since the presence of fillers has a clear effect on mechanical properties acquired during the test [10]. The quality monitoring of processes such as injection molding could be significantly improved by the introduction of FIMEC test [14], suitable to investigate critical zones of molded parts (e.g., in proximity of the weld lines) whose strength can be affected by fillers concentration. As regards CFRP, Abisset et al. [15] described the evolution of the damage during static indentation on plates. Non-destructive techniques, such as ultrasonic scanning and X-ray computed tomography, were compared to evaluate the damage evolution within the plates at different load levels. However, in the pertinent literature, no correlation between an indentation and moisture absorption was conducted. For the first time in [2] the slope of the linear trend of FIMEC curves was related to the local stiffness, demonstrating that macro indentation can be used to evaluate the water uptake effect. If compared to other standard methods, such as mechanical tests (tensile and bending tests) FIMEC is a non-destructive test, which can also be applied to an actual structure without the need for cutting-out a specimen. In [2] the authors performed both FIMEC test and short-beam shear tests in order to investigate the global degradation in the CFRP. The matrix Young’s modulus and the flexural modulus of the composites were compared. A good correlation between the results was observed, showing that FIMEC indentation test can be adopted to study the moisture effect. Moreover, the reduction of the stiffness being higher than the shear stress in almost every condition, it is possible to say that the FIMEC test is more sensible than the short-beam test [2]. If compared to other non-destructive techniques, such as ultrasonic scanning and X-ray computed tomography, FIMEC allows the evaluation of the damage evolution by using simple equipment and procedure.

In the present study, the innovative application of FIMEC in the field of water uptake characterization was investigated, aiming at verifying the ability of the testing procedure to discriminate among different surface conditions. Two typologies of coating for water absorption reduction were considered, so as to define different scenarios for the validation of the proposed procedure. FIMEC results obtained for uncoated samples and coated by water repellent or hydrophobic resins were compared. In particular, siloxane and silicon-based coatings were formulated and applied on a part of samples to reduce the moisture absorption of the material. The choice was determined by the protective performance exhibited by these coatings on metals. In particular, the latter type is widely used as protective barriers for metal substrates, and the resulting coatings have been deeply characterized in terms of morphology, scratch resistance, and tribological performance [16,17]. In the present study a formulation was defined for composite substrates. 

An industrial product based on a hydroxy-repellent siloxane, Silomur, was used. The siloxanes are man-made saturated silicone-oxygen hydrides and after polymerization give rise to the silicones. Siloxanes are used in many commercial and industrial applications due to the compounds’ hydrophobicity, low thermal conductivity, and high flexibility. Siloxanes are molecules with an oxygen–silicon backbone (Si–O–Si) where each Si atom carries two organic groups, mostly methyl, ethyl, or phenyl groups. Depending on their molecular weight, siloxanes can be characterized as linear or cyclic volatile methyl siloxanes, polydimethylsiloxanes (PDMS), or polyethermethylsiloxanes (PEMS). Cyclic siloxanes are produced by chemical synthesis, starting with the hydrolyzation and polymerization of dichlorodimethyl silane. Silicone elastomers, or silicone rubbers, are formed from fluid siloxanes during the process of vulcanization via cross-linking agents, which mediate the formation of cross-links between the linear polymers. Linear and cyclic volatile methylsiloxanes are man-made chemicals with no natural source. Cyclic siloxanes are mainly used as intermediates for the production of higher-molecular-weight siloxanes or directly as fluids.

Linear polyorganosiloxanes can be prepared from cyclic organosiloxanes by equilibrating ring-opening polymerization, which may be initiated by both anionic and cationic catalysts. Octamethylcyclotetrasiloxane is the preferred starting material, while other siloxanes are used for chain end-stopping or for producing copolymers. [18,19]

The silicon-based coating was obtained by using Silikopon EF, which belongs to the general class of Silikopon. The Silikopon combines the properties of the widely applicable epoxide resins with those of silicone resins. The modification with silicones has been a subject of intensive research aimed at combining the advantages offered by epoxy resins with those of silicones. It has been shown that modification of epoxy resins with carbofunctional silanes, siloxanes, and polysiloxanes leads to significant improvements in the overall properties of the cross-linked system and it offers new possibilities for their use in the coating industry, as composite and nanocomposite materials. It has been shown that these materials possess a valuable combination of properties, such as improved mechanical properties, thermal stability, and resistance to oxidation, weathering, and chemicals [20,21]. Good adhesion properties and improved corrosion protection make these materials highly advantageous for protective coatings industry. Although significant scientific efforts have been made to synthesize various silicone-epoxy hybrid resins [22], only a limited number of these hybrids became commercially available, generally as protective coatings for concrete and metal surfaces. Among them, the Silikopon products from Evonik Industries are aimed for topcoat and heat-resistant coating formulations [23]. 

Silikopon EF is a silicone-epoxide hybrid binder with silicone content of 50% for use in topcoats in general industrial and maritime applications. Silikoponr EF is used as a binder for ultra-high solids applications in corrosion protection coatings for steel, coatings for wood and concrete and maritime applications such as biocide-free, easy-to-clean coatings particularly above the water line. The special feature of the chemical crosslinking of Silikopon EF lies in the dual cure mechanism at room temperature: the nucleophilic opening of the epoxide ring (by the amine) and the hydrolysis/condensation reaction of the alkoxy groups. Both reactions occur in situ. The curing agents are aminosilanes whose amine groups react with the epoxide groups. The three alkoxy groups react in the presence of water or moisture with the free alkoxy group of the silicone resin by hydrolysis/condensation. This “double crosslinking” allows the positive properties of organic and inorganic polymers to be combined in a new class of binders. Because of the high crosslink density, these coatings have a high dirt repelling effect [24].

Some authors have studied systems based on Silikopon EF resin and the effect of the ATPES curing agent on the basic properties of the resin. They reported that the solvent and chemical resistance as well as the hardness and adhesion properties of the cured resin samples were excellent [23].

Uncoated samples and others coated by using the product described, Silomur and Silikopon, were analyzed in terms of moisture absorption.

The characterization procedure based on weight measurement and FIMEC test, proposed in the present study, was found to evaluate the local reduction of the stiffness of CFRP plates, after different ageing in water. The ability of the FIMEC test to discriminate between various surface conditions of the same material (coating) was established.

Moreover, IR analysis was used as supporting data. The analysis of infrared spectra (acquired in the range 4000–600 cm^−1^) allowed the identification of the molecules in a sample, and the evaluation of their concentrations. The spectrum of each sample was compared to the reference spectrum, in terms of peak position, height, and width. A fast and easy measurement procedure was defined. 

The presence of water was evaluated by analyzing the peaks of the stretching band of the -OH group, visible in proximity of the 3400 cm^−1^ value of the wavenumber. The moisture absorption can be related to the spectra, as the peak tends to rise due to the water uptake phenomenon, as explained in the pertinent literature [25,26].

## 2. Experimental

### 2.1. Materials 

The investigated samples were CFRP plates 4 mm in thickness, autoclave cured, for aeronautical use. T400 carbon fibre reinforced according to the following layup: four external plies of fabric 193 g/m^2^ on each face, with 50% fibre in the warp direction, and sixteen internal unidirectional plies of 145 g/m^2^, placed at ± 45°. The matrix was HMF 934 epoxy resin. The fibre volume ratio was about 60%, corresponding to a density of 1540 kg/m^3^.

Some samples were coated by the dip-coating process, using commercially available aliphatic silicone-epoxy hybrid resin (silicon-based) and siloxane formulations. Silikopon EF (Evonik Industries, Pandino, Cremona, Italy), with epoxide equivalent weight of 450 g/mol, and the siloxane water repellent Silomur (CAP Arreghini) were used. Silikopon EF has a viscosity of 1500 mPas at 25 °C and concentration of 98%. One type of bifunctional organosilane was used as hardener: 3-aminopropyltriethoxysilane (Dynasilan APTES) with amine hydrogen equivalent weight of 110.5 g/mol. The selected organosilane was supplied by Evonik Industries, Germany. The catalyst Dibutyltin dilaurate (DBTDL) was purchased from Sigma Aldrich (Milano, Italy). All materials were used without any purification.

The hydrophobic silicon epoxy resin (binder) was mixed to the hardener amino-propyl triethoxy silane with 2:1 mixing ratio. Dibutyltin dilaurate (DBTDL) was used as a catalyst at 2 wt% to increase the curing speed. 

The surface of CFRP plates was opportunely cleaned before being coated. Subsequently, the substrate were dipped vertically in resins and withdrawn after 2 s with a fixed speed of 2 mm/s. Finally, the samples were dried for 48 h at room temperature (24 °C and 50% of relative humidity).

### 2.2. Characterization 

**Figure 1:**
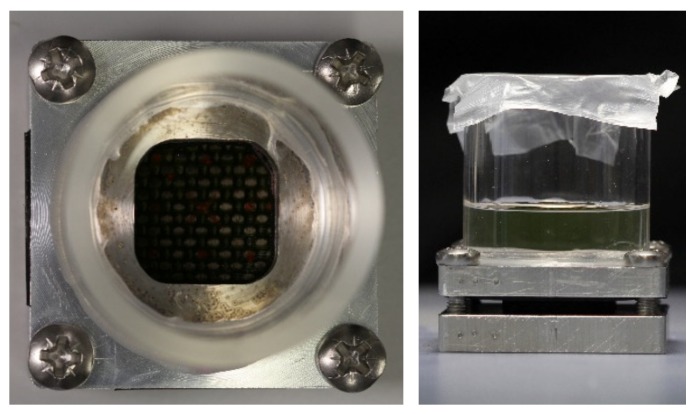
Sample holder for ageing in water.

Before testing, the specimens were placed at 50 °C for 24 h to dry them and standardize the initial test conditions. When reached the room temperature, the dried samples were put in the holder in Figure 1. It was used to place distilled water at 24 °C onto a surface of the sample, avoiding the edge effect. The holders were placed in a climatic chamber at controlled temperature at 24 °C, where the water uptake process took place. The water uptake was evaluated according to the ASTM D570 specifications [19]. The specimen weight was recorded every 24 h up to 336 h. The water gain percentage was determined. A subsequent phase of drying was performed before the characterization. 

The FIMEC indentation tests were conducted after each step of ageing in water by a Universal Test machine (Insight 5 by MTS) equipped with a 2.5 kN load cell and WC (tungsten carbide) cylindrical indenter (1 mm in diameter), under quasi-static conditions (speed 0.1 mm/min). Each ageing time required a sample, since the indentation could affect the subsequent water absorption. Nine repetitions of FIMEC tests were performed on each sample, obtaining load vs. penetration curves. Many parameters can be extracted by these curves to analyze different materials on a comparative base such as the load value at fixed penetration depth or the slope in a given range of depths. In particular, the slope of the linear trend is considered being related to mechanical stiffness of the indented material [27]. As shown in [27], the FIMEC method allows the determination on a local scale of stiffness and yield stress as obtained in standard tensile tests. 

In the field of composite materials, the loading condition of the indentation test refers to properties of the composite in the direction normal to the fibers; therefore, this test can be used for evaluating the transverse elastic modulus of the composite [2]. 

In the present study, the stiffness of the tested samples, as estimated by the slopes of the load vs. penetration curves, was subsequently related to the water uptake phenomenon. 

To evaluate the efficiency of the coatings, IR transmission spectra were obtained to evaluate the signals relating to the absorption of water. FTIR (Fourier transform infrared spectroscopy) spectra (FT–IR Nexus spectrometer) and the Omnic 6 were recorded in transmittance using a Thermo Nicolet software for a spectral range of 4000–600 cm^−1^ with a resolution of 2 cm^−1^ and an acquisition rate of 64 scans min^−1^. The spectra were processed by correcting the baseline and the CO_2_ signal and normalizing against the highest peak.

**Figure 2:**
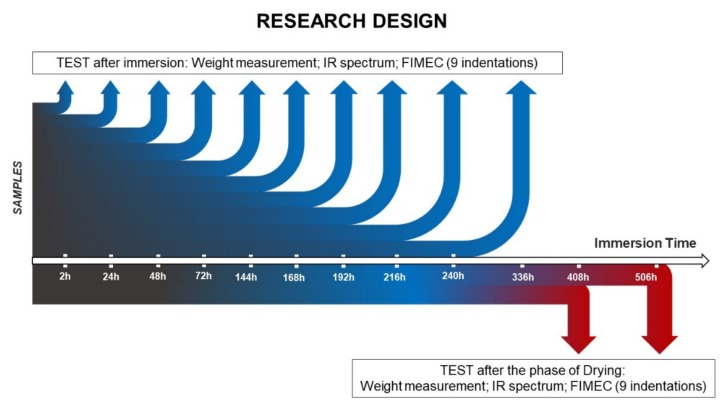
Experimental design.

The experimental design is shown in Figure 2.

## 3. Results and Discussion 

FIMEC tests were performed before immersion to analyze coated and uncoated samples on a comparative base. The slope of the linear trend is shown in Table 1. 

FIMEC tests were performed in parallel with both IR analysis and measures of weight of the specimens to evaluate the performance of uncoated and coated CFRP when water absorption takes place. The matrix used for the investigated CFRP plates was epoxy resin. In fact, though the good mechanical and chemical properties, and excellent adhesion to fibers, a challenging problem for epoxy of resin is the water uptake values, which set a limit on the utilization in the aerospace market [26].

As an indicator of the water uptake, $W_{ut}$, the percentage difference in the weight of the material, normalized with respect to the initial weight, was chosen at different instants. $W_{ut}$ represents the variation of water uptake over time, it was calculated at the initial time and then every 24 h of liquid exposure, up to 336 h through the equation in [28]:

(1) $$W_{ut}=\frac{P_{t\;\;}-P_{0}}{P_{0}}\times 100$$

where *P_t_* and *P_0_* represent the weights of the specimen at time *t* and at the initial time, respectively. The test conditions and the average Wut percentage during the water uptake and the driyng are reported in Table 2.

**Figure 3:**
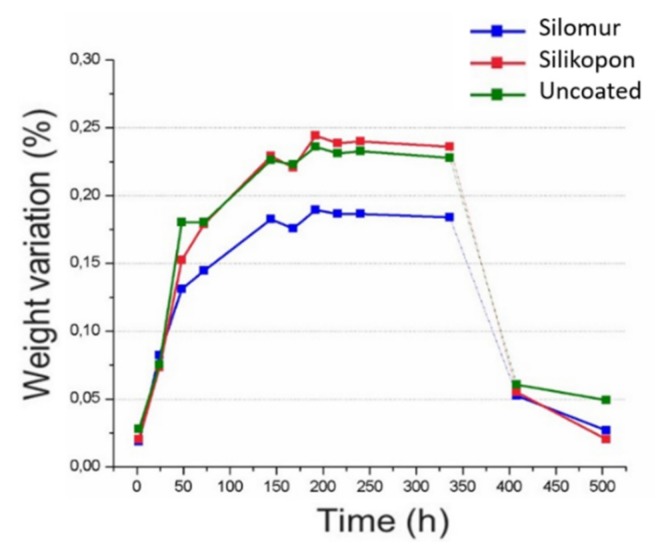
Water uptake according to ASTM D570 specifications.

Figure 3 shows the moisture absorption of the three types of samples (uncoated, coated by Silomur and Silikopon) as a function of time. The continuous lines connect experimental points related to the various step of ageing in water; the dotted lines indicate a phase of drying in the oven, which returned the specimens to the initial conditions. As expected, an increase in weight was due to the water absorption, and a plateau was reached within 200 h. 

Enhanced characteristics were resulted in the samples coated by using siloxane-based formulation (Silomur). A lower increase in weight was recorded for each immersion time if compared to the other two typologies of samples (uncoated and Silikopon). These latter samples exhibited a similar behavior, the curves are almost overlying. Therefore, Silikopon treatment was not efficient, and after 200 min water could penetrate in the interface between coating and substrate.

From the load vs. penetration curves obtained by FIMEC tests, the slope of the initial linear trend was extracted, according to the experimental procedure in [2]. This slope was considered for samples comparison, being related to local mechanical stiffness of the indented material. A reduction of the slope could be ascribed to the water uptake effect. 

**Figure 4:**
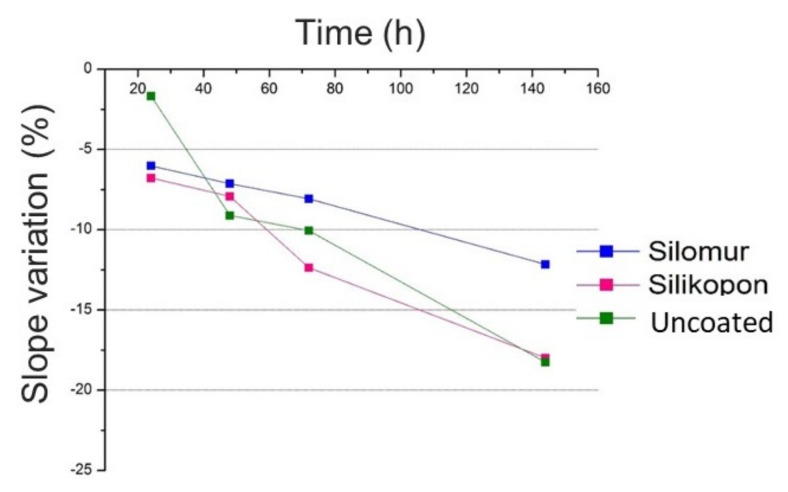
FIMEC slope as a function of ageing time.

The reduction of the FIMEC slope is presented in Figure 3. The average values of slope from the nine repetitions were considered to obtain the graph. It stated that the FIMEC test is suited to the evaluation of the water uptake in composite materials, since a decreasing trend was evident. Moreover, it is practical to discriminate various surface conditions of the same substrate, in fact a different slope was observed for the three typologies of sample (uncoated, coated by Silomur and by Silikopon). A different behavior was observed for samples coated by Silomur, the ones that absorbed the water in less concentration (Figure 4). 

**Figure 5:**
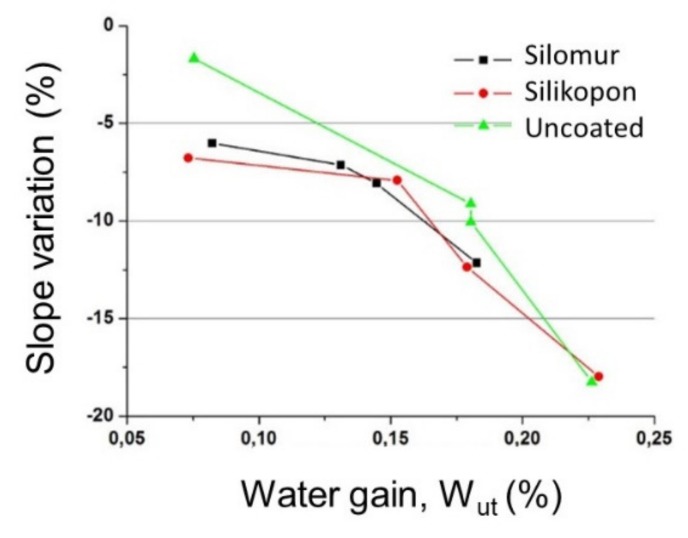
FIMEC slope as a function of water uptake.

In Figure 5 the evolution of local mechanical properties was analyzed as a function of the water uptake. A decrease of FIMEc slope according to increasing $W_{ut}$ was found for all the compared samples, but it was higher for the uncoted ones.

The presence of water inside the CFRP plates was evaluated by IR transmission spectra. The existence of water can be in terms of: molecules bound to specific sites as “bound water” and clustered in microvoids as “free water.” The amount of bound water mainly depends on the polarity of molecules while the amount of free water is determined by network structure [26]. In particular, the presence of water was evaluated in this study by the signal relating to the stretching band of the -OH group visible in proximity of the 3400 cm^−1^ range of the wave number. The broad band was assigned to O-H stretching of hydroxyl groups according to the pertinent literature [25].

**Figure 6:**
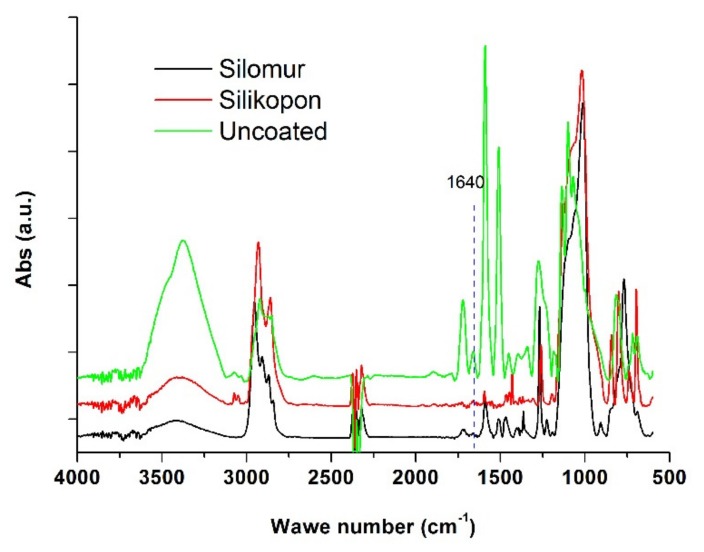
IR spectrum of Silomur, Silikopon EF, and uncoated samples at the initial time.

As Figure 6 shows, another very low-intensity signal was observed at 1640 cm^−1^, it can be related to the bending vibration of the -OH group. While the broad band in the range 3400–3600 cm^−1^ is assigned to the stretching vibration of hydroxyl groups, other relevant absorption bands are present besides these. Around 2900 cm^−1^ the signals observed from the spectra are attributable to the alkane groups. The signals around 1590, 1100, and 800 cm^−1^ are referred to the NH and NH_2_ groups, relating to the amine hardeners of the epoxy resin. The strong band positioned in the 1100–1000 cm^−1^ range, is associated with Si-O-C stretching vibrations in alkoxysilanes. The band appearing at 1130 cm^−1^ is associated with the developing of Si-O-Si linkages, characteristic for cured hybrid silicone-epoxy resins. Furthermore, the signals relating to the aromatic groups with low intensity absorption around 1500 cm^−1^, attributable to the aromatic structure of the carbon fibers, are visible.

**Figure 7:**
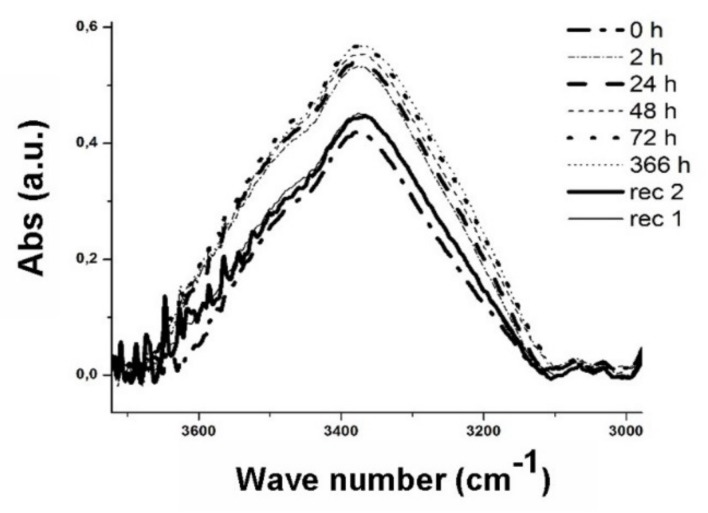
IR spectrum of the -OH signal for uncoated samples at different ageing time.

**Figure 8:**
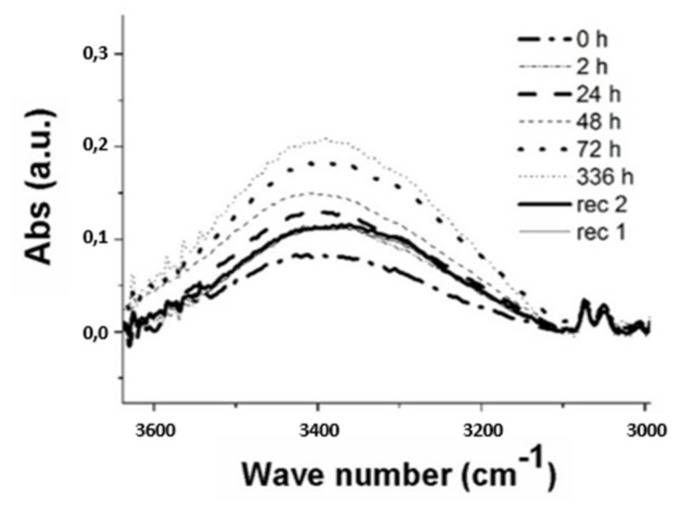
IR spectrum of the -OH signal for samples coated by Silicopon at different ageing time.

**Figure 9:**
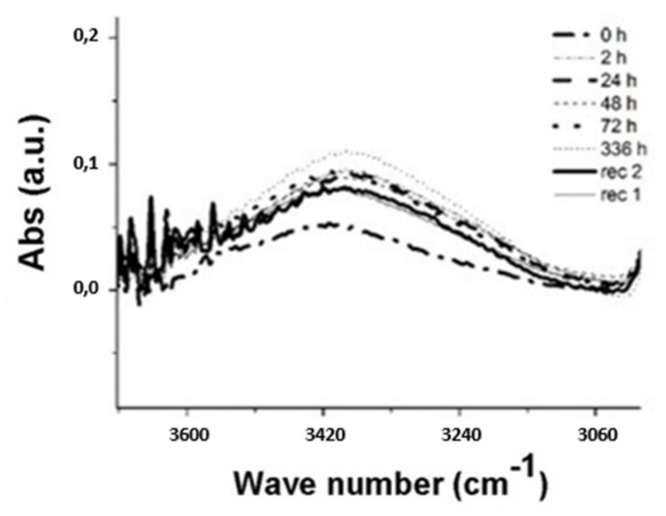
IR spectrum of the -OH signal for samples coated by Silomur at different ageing time.

Figure 7, Figure 8, Figure 9 show the spectra obtained at the peak relative to the stretching band of the -OH group. On the other hand, the absorptions related to –OH bending vibration have been neglected because they are smaller than the first ones.

The spectra show that as the water exposure (hours) increase, the peak tends to rise due to the water uptake phenomenon. A decrease in absorbance is obtained after 336 h, being the three types of samples subjected to the process of recovery of the initial conditions by means of a vacuum oven (pressure −1 bar) at 35 °C. After the water gain steps the two phases of drying (recovery), rec 2 and rec 1, were performed at 408 h and 504 h, respectively. 

It is noted that the recovery treatment is not able to re-establish the initial conditions (t = 0 h) due to the water trapped in the structure.

The curves after 72 h of exposure were omitted because the saturated samples no longer underwent further significant variations in the absorption peak. A quantitative measure of the water uptake phenomenon was obtained by calculating the area under the peaks as the treatment time increases.

The percentage growth values of the areas were compared with the respective percentage increments of the sample weights to develop a correlation.

**Figure 10:**
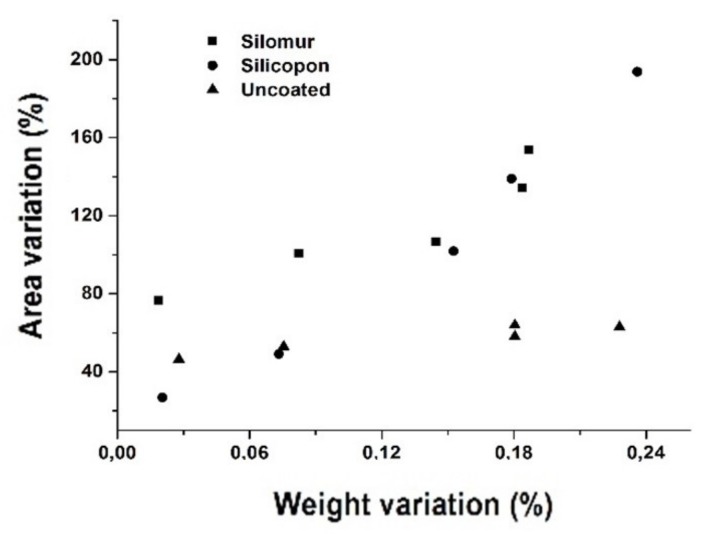
Correlation graphs of percentage variations of areas and weights for uncoated samples and coated by the Silomur or Silicopon.

Figure 10 shows a linear correlation between the area and weight variations undergone by the coated samples, as the water absorption increases the area of the -OH peak increases, in turn increasing the exposure time to the liquid, as reported in Table 3.

Due to the different amplitudes of the signals, the whole area was considered in order to carry out a semi-quantitative analysis. The calculated areas related to the absorptions of the -OH group are smaller for the Silomur sample and higher in the case of the uncoated sample, in accordance with the data obtained by the FIMEC test. 

The proposed method allows to inversely correlate the mechanical properties, in terms of resistance to indentation, with the area subtended by the peak typical of water absorption. It is a promising procedure to study the water uptake phenomenon of high-performance materials, such as CFRP.

## 4. Conclusions 

The effect of moisture absorption on the mechanical properties of coated and uncoated CFRP was investigated. Laminates for aeronautical use, autoclave-cured, composed of epoxy resin and carbon fibers, were coated by silicon-based and siloxane formulations, which affected the moisture absorption differently. 

The FIMEC indentation test was adopted to study the reduction of the mechanical characteristics of the three typologies of samples (uncoated, coated by Silomur and coated by Silikompon), under different water ageing conditions. In particular, the FIMEC loading condition allowed the authors to investigate the properties of the composite in the direction normal to the fibers. The evolution of local mechanical properties is reported as a function of the water uptake.

A reduction of composites property was stated by the reduction of the initial slope (N/mm) of FIMEC curves, related to the local stiffness of the indented materials. Different trends were observed according to the coatings, resulting in the ability of the FIMEC test to discriminate among various surface conditions of the same material. The proposed test represents an accurate tool for assessing the water uptake, moreover, it is a non-destructive procedure and performed in a short time. 

The procedure based on FIMEC charaterization and analysis of IR sprectra allows to inversely correlate the mechanical properties and the area subtended by the peak typical of water absorption (-OH group). It is a promising procedure to study the water uptake phenomenon of high-performance materials, such as CFRP.

## Figures and Tables

**Table 1:** FIMEC slope before immersion.

Test Repetition	SILOMUR	Slope (N/MM) SILIKOPON	UNCOATED
**1**	4448.3	3724.5	4850.9
**2**	4197.4	3817.2	4445.7
**3**	4653.1	3982.4	4352.8

**Table 2:** Test conditions and average water uptake percentage.

	Average Water Uptake, Wut (%)		
Immersion Time (h)	Silomur	Silikopon	Uncoated
0	–	–	–
2	0.0206	0.0187	0.0279
24	0.0734	0.0824	0.0754
48	0.1526	0.1312	0.1804
72	0.1790	0.1447	0.1804
144	0.2291	0.1827	0.2263
366	0.2360	0.1840	0.2279
408 (*)	0.0553	0.0526	0.0607
504 (*)	0.0206	0.0268	0.0492

(*) dried at 35 °C in vacuum (−1 bar).

**Table 3:** Amplitude of the -OH peak.

Time (h)	SILOMUR	Peak Amplitude (a.u.) SILIKOPON	UNCOATED
0	14.199	22.498	108.141
2	25.069	28.527	158.289
24	28.485	33.537	165.324
48	25.848	45.419	170.944
72	29.326	53.754	177.222
144	30.126	55.701	162.4
168	40.413	57.415	172.645
192	39.72	55.799	172.121
216	35.998	57.439	174.129
240	42.642	54.188	169.178
336	33.256	66.106	176.241
408	28.381	33.48	131.399
504	24.123	34.885	130.678
